# Efficacy Evaluation of Luliconazole‐Loaded Nanostructured Lipid Carriers in Treatment‐Resistant Dermatophytosis: A Randomized Clinical Trial

**DOI:** 10.1111/myc.70196

**Published:** 2026-06-08

**Authors:** Robab Ebrahimi Barogh, Seyed Ali Jeddi, Ghasem Rahmatpour Rokni, Hossein Mohammad Taghi Fam, Abbas Raeisabadi, Zahra Yahyazadeh, Seyyed Mobin Rahimnia, Pedram Ebrahimnejad, Eisa Nazar, Nasim Gholizadeh, Iman Haghani, Mohsen Nosratabadi, Armaghan Kazeminejad, Javad Javidnia, Mohammad Taghi Hedayati, Jacques F. Meis, Ahmed Al‐Harrasi, Mahdi Abastabar, Abdullah M. S. Al‐Hatmi

**Affiliations:** ^1^ Department of Medical Microbiology, Immunology, Parasitology, School of Medicine Ardabil University of Medical Sciences Ardabil Iran; ^2^ Student Research Committee Mazandaran University of Medical Sciences Sari Iran; ^3^ Department of Medical Mycology, School of Medicine Mazandaran University of Medical Sciences Sari Iran; ^4^ Department of Dermatology, Faculty of Medicine Mazandaran University of Medical Sciences Sari Iran; ^5^ Pharmaceutical Sciences Research Centre, Haemoglobinopathy Institute Mazandaran University of Medical Sciences Sari Iran; ^6^ Department of Pharmaceutics, Faculty of Pharmacy Mazandaran University of Medical Sciences Sari Iran; ^7^ Psychiatry and Behavioral Sciences Research Center, Addiction Institute Mazandaran University of Medical Sciences Sari Iran; ^8^ Invasive Fungi Research Center, Communicable Diseases Institute Mazandaran University of Medical Sciences Sari Iran; ^9^ Department of Laboratory Sciences Sirjan School of Medical Sciences Sirjan Iran; ^10^ Department of Dermatology, Antimicrobial Resistance Research Center, Communicable Diseases Institute Mazandaran University of Medical Sciences Sari Iran; ^11^ Department of Medical Microbiology, Radboudumc and Centre for Expertise in Mycology Radboudumc/CWZ Nijmegen the Netherlands; ^12^ Institute of Translational Research, Cologne Excellence Cluster on Cellular Stress Responses in Aging‐Associated Diseases (CECAD), Excellence Center for Medical Mycology (ECMM), University of Cologne Cologne Germany; ^13^ Natural and Medical Sciences Research Center University of Nizwa Nizwa Oman

**Keywords:** antifungal resistance, clinical trial, dermatophytosis, luliconazole, nanostructured lipid carriers, *trichophyton indotineae*

## Abstract

**Background:**

Dermatophytosis is a common superficial fungal infection that is increasingly complicated by terbinafine (TRB)‐resistant strains, especially *Trichophyton indotineae*. Conventional antifungals often show limited efficacy against resistant isolates, highlighting the need for novel treatments. This study evaluated the efficacy and safety of nanostructured lipid carrier–loaded luliconazole (NLC‐LCZ) gel in TRB‐resistant dermatophytosis.

**Materials and Methods:**

In a randomized, clinical trial conducted at Touba Specialty Clinic, Sari, Iran, forty‐eight adults with confirmed TRB‐resistant dermatophytosis were assigned to oral itraconazole (ITZ), topical luliconazole, NLC‐LCZ gel, or ITZ + NLC‐LCZ gel for 4 weeks (*n* = 12 each). The primary endpoint was complete recovery (clinical and mycological), while secondary endpoints included lesion size, itch, inflammation, Dermatology Life Quality Index (DLQI), and safety.

**Results:**

Complete recovery occurred in 83.3% of ITZ + NLC‐LCZ, 75% of NLC‐LCZ, 58.3% of LCZ, and 0% of ITZ alone. LCZ and NLC‐LCZ had lower MIC_50_/ MIC_90_ values (0.016 μg/mL) compared with ITZ (MIC_90_: 8 μg/mL) and TRB (MIC_90_: 4 μg/mL). DLQI improved most in NLC‐LCZ (score: 2) and ITZ + NLC‐LCZ (score: 3) groups (*p* < 0.001). Adverse events were mild and did not require discontinuation.

**Conclusions:**

These findings indicate that NLC‐LCZ enhanced antifungal efficacy and quality of life in TRB‐resistant dermatophytosis, and that combination therapy with ITZ + NLC‐LCZ yielded the highest recovery, representing a promising strategy against resistant infections.

## 1 Introduction

Dermatophytosis is a common fungal infection affecting the skin, hair, and nails, posing a significant global public health concern [1]. This condition is caused by dermatophyte fungi, which are capable of infecting both humans and animals, leading to a spectrum of inflammatory skin disorders [2]. Globally, superficial fungal infections affect an estimated 20%–25% of the population [3].

Conventional antifungal therapies, including azoles and allylamines, have been widely used to manage dermatophytosis. However, the increasing prevalence of resistant strains has compromised treatment efficacy, contributing to higher morbidity and economic burden [4, 5, 6]. Among the most prominent dermatophyte species responsible for these infections are *Trichophyton rubrum* and *Trichophyton mentagrophytes*. Recently, a newly identified subspecies, *Trichophyton indotineae*, has emerged, exhibiting high levels of resistance to terbinafine, the first‐line treatment for dermatophytosis [1, 7].

Luliconazole (LCZ) is a topical imidazole antifungal with strong activity against dermatophytes. Its antifungal effect is mainly related to inhibition of lanosterol 14α‐demethylase, a key enzyme involved in ergosterol biosynthesis. Interference with this pathway disrupts the fungal cell membrane and ultimately inhibits fungal growth. In clinical practice, LCZ has been used widely for the treatment of superficial dermatophyte infections such as tinea corporis, tinea cruris, and tinea pedis, where it has shown good therapeutic efficacy as a topical agent [8].

Recurrent infections, particularly onychomycosis, highlight the limitations of existing treatments. Poor penetration of antifungal agents into keratin‐rich tissues often results in therapeutic failure [9, 10]. Consequently, there is a pressing need for advanced drug delivery systems to improve efficacy while minimizing adverse effects [10, 11]. Nanostructured lipid carriers (NLCs) have emerged as a promising strategy; however, their application for delivering LCZ, a potent antifungal agent, remains underexplored [11, 12, 13, 14].

In a previous study, luliconazole‐loaded NLC (NLC‐LCZ) was formulated and demonstrated superior in vitro activity against 62 resistant fungal isolates, including *Trichophyton*, *Aspergillus*, *Fusarium*, and *Candida*, identified using molecular methods [11]. These findings provide a solid foundation for further clinical evaluation of NLC‐LCZ, offering a potential novel approach for managing treatment‐resistant dermatophytosis. The present study therefore aimed to evaluate the potential of NLC‐LCZ for improving antifungal efficacy against resistant dermatophyte infections.

## 2 Materials and Methods

### 2.1 Clinical Study Design

This randomized clinical trial was conducted in 2024 at Touba Specialty Clinic, Sari, Iran, and enrolled patients with terbinafine (TRB)‐resistant dermatophytosis. The diagnosis was confirmed through positive microscopic examination, fungal culture, followed by antifungal susceptibility testing. Because the interventions were administered via different routes (oral itraconazole versus topical formulations), blinding of participants, clinicians, and laboratory personnel was not feasible. Accordingly, the study was designed as an open‐label randomized clinical trial, in which all parties were aware of the assigned treatment. The study protocol was approved by the Ethics Committee of Mazandaran University of Medical Sciences (IR.MAZUMS.REC.1403.048) and registered in the Iranian Registry of Clinical Trials (IRCT20240711062393N1). The full trial protocol is available on the IRCT website (https://www.irct.ir). Written informed consent was obtained from all participants in accordance with ethical guidelines.

### 2.2 Patient Selection and Treatment Groups

**FIGURE 1:**
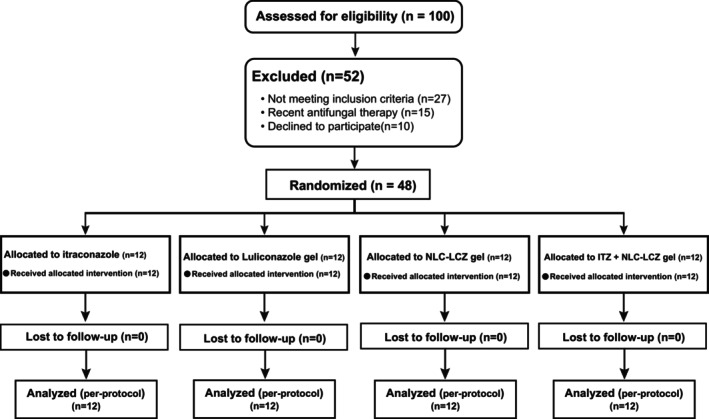
CONSORT flow diagram of the clinical trial.

The study included adults aged 18–55 years with TRB‐resistant dermatophytosis caused by *T. indotineae*, confirmed via microscopic examination, fungal culture, antifungal susceptibility testing, and molecular methods. Clinical manifestations included pruritus, erythema, annular lesions, and vesicular eruptions that persisted despite conventional antifungal treatment. Exclusion criteria were pregnancy, lactation, use of immunosuppressive agents, recent exposure to known allergens, and administration of antibiotics or antifungal drugs within 2 weeks prior to enrollment. A total of 100 patients were screened for eligibility. Fifty‐two patients were excluded: 27 did not meet the inclusion criteria, 15 had received systemic or topical antifungal therapy within the previous 2 weeks, and 10 declined participation. The remaining 48 eligible patients were enrolled after providing written informed consent. The flow of participants through the stages of screening, randomization, allocation, follow‐up, and analysis is summarized in the CONSORT flow diagram (Figure 1). Participants were randomly allocated to four groups (12 patients each): a control group receiving itraconazole (ITZ) alone, and three treatment groups receiving (i) LCZ gel, (ii) NLC‐LCZ gel, or (iii) oral ITZ combined with NLC‐LCZ gel. Topical gels were applied once daily following standardized instructions. The total treatment duration was 4 weeks. The LCZ and NLC‐LCZ formulations were developed in collaboration with the School of Pharmacy at Mazandaran University of Medical Sciences and were distributed to patients for the study. Participants were instructed to return to the clinic if they experienced irritation or worsening of symptoms. Randomization was carried out manually using a table of random numbers to ensure balanced allocation across the four treatment groups. This procedure was performed by a research team member who was not involved in patient enrollment or outcome assessment. Allocation concealment was maintained using sequentially numbered, opaque, sealed envelopes that were opened only after participant inclusion. Because the interventions differed in route and form of administration, blinding was not feasible; thus, the study was conducted as an open‐label trial. However, the allocation procedure was standardized to minimize potential selection bias. In the control group, itraconazole was administered orally at a dose of 100 mg twice daily (total daily dose 200 mg) for 4 weeks. In the combination group, the same oral itraconazole regimen (100 mg twice daily) was combined with topical NLC‐LCZ gel applied once daily for 4 weeks.

### 2.3 Clinical and Efficacy Assessment

All randomized participants completed the four‐week treatment and follow‐up assessments, and no participants were lost to follow‐up. Therefore, all 48 patients were included in the final per‐protocol analysis. Patients were evaluated at baseline and at 2 and 4 weeks after treatment initiation. Clinical severity was assessed using a predefined semi‐quantitative scoring system based on previously described dermatological assessment methods [15]. Key parameters, including lesion size, degree of inflammation, pruritus, scaling, and patient satisfaction, were each graded on a 6‐point scale ranging from 0 (“none”) to 5 (“very severe/extensive”). The detailed criteria for this scoring system are provided in Table S1.

Complete recovery was defined as the simultaneous presence of clinical cure (resolution of erythema, scaling, inflammation, and pruritus) and mycological cure (negative direct microscopy and culture). Patients who showed clinical improvement but remained mycologically positive, or those with residual mild pruritus despite improvement of lesions, were categorized as partial responders rather than complete cures. Patients were additionally followed beyond the 4‐week treatment period to monitor relapse and adverse events; however, these follow‐up data were not included in the formal efficacy analysis.

### 2.4 Laboratory Procedures

Skin samples were assessed both before and after end of treatment. The samples were cultured under sterile conditions on Sabouraud dextrose agar (SDA, Merck, Darmstadt, Germany) and incubated at 30°C for 2 weeks. Cultures were also subcultured on Sabouraud chloramphenicol cycloheximide (SCC) agar to facilitate selective isolation of dermatophytes. A drop of 10% potassium hydroxide was used for direct microscopic examination. DNA extraction was performed from fresh colonies cultured on SDA following a previously established protocol [16, 17]. The internal transcribed spacer (ITS) rDNA region was amplified using universal primers ITS1 (5′‐TCCGTAGGTGAACCTGCGG‐3′) and ITS4 (5′‐TCCTCCGCTTATTGATATGC‐3′) [16]. The PCR products were sequenced, and the obtained sequences were compared with reference sequences in the GenBank database using the BLAST algorithm to confirm the identification of *Trichophyton indotineae*.

### 2.5 In Vitro Antifungal Susceptibility Testing

Antifungal susceptibility testing was conducted using broth microdilution techniques as outlined in the Clinical and Laboratory Standards Institute (CLSI) M38‐A3 guidelines. Stock solutions of LCZ (Nihon Nohyaku Co.), NLC‐LCZ, TRB (TRB, Sigma‐Aldrich, St. Louis, MO, USA), and ITZ (Janssen Pharmaceutica, Beerse, Belgium) were prepared in dimethyl sulfoxide (DMSO). The concentrations of drugs in the wells were as follows: ITZ ranged from 0.016 to 16 μg/mL, TRB from 0.004 to 4 μg/mL, and both LCZ and NLC‐LCZ from 0.001 to 1 μg/mL. MICs were determined after 72 h of incubation at 35°C and defined as the lowest concentration resulting in ≥ 80% growth inhibition [18]. Quality control strains included *Candida parapsilosis* ATCC 22019, *Candida krusei* ATCC 6258, and *Aspergillus flavus* ATCC 2004304. All tests were conducted in duplicate to ensure accuracy and reliability.

In this study, TRB resistance was defined based on both clinical and microbiological criteria. Clinical resistance referred to persistent or progressive dermatophytosis despite adequate prior terbinafine therapy. Microbiological resistance was determined by elevated TRB MIC values obtained using the CLSI M38‐A3 broth microdilution method. Because formal CLSI clinical breakpoints for TRB against dermatophytes have not yet been established, elevated MIC values (≥ 4 μg/mL) were considered indicative of resistance based on previously published studies [18, 19].

### 2.6 Statistical Analysis

Data were analysed using SPSS version 16. Continuous variables were expressed as mean ± SD or median, as appropriate. Categorical outcomes (e.g., complete recovery, adverse events) were analysed using Chi‐square tests. Continuous outcomes (e.g., symptom severity, DLQI, lesion size, pruritus, and inflammation scores) were compared using *t*‐tests or one‐way ANOVA, followed by appropriate post hoc tests (Tukey's test for parametric data or Dunn's test with Bonferroni correction for non‐parametric data). A *p* < 0.05 was considered statistically significant. All randomized participants completed the 4‐week treatment and follow‐up assessments. Therefore, outcome analyses were performed on a per‐protocol basis including all participants who completed the study.

Sample size was calculated from prior data [20], requiring 11 patients per group; with a 10%–20% dropout rate, 12 patients per group (48 total) were enrolled. Sample size estimation was based on preliminary data from a previous study. The assumptions included an expected effect size of 0.9 for the reduction in clinical severity scores, a standard deviation of 1.1, a two‐sided alpha of 0.05, and a statistical power of 80%. Under these assumptions, 11 participants per group were required. Allowing for a 10%–20% dropout rate, we enrolled 12 patients in each group. Given the relatively small sample size, the study carries an inherent risk of type II error in detecting smaller between‐group differences. Therefore, the present trial should be considered an exploratory pilot study intended to generate preliminary effect estimates for future large‐scale randomized trials.

## 3 Results

### 3.1 Isolates Identification

All 48 dermatophytes isolates were confirmed as *Trichophyton indotineae* by DNA sequence analysis of the internal transcribed spacer (ITS rDNA) region. Representative nucleotide sequences obtained in this study were deposited in GenBank under accession numbers PV628869–PV628904 (Seq1 to Seq36).

### 3.2 In Vitro Antifungal Susceptibility

The antifungal susceptibility of 48 *Trichophyton indotineae* isolates was assessed against four antifungal agents: NLC‐Luliconazole (NLC‐LCZ), LCZ, Itraconazole (ITZ), and TRB. Table 1 presents the minimum inhibitory concentrations (MICs), MIC ranges, MIC_50_, MIC_90_, geometric mean (GM), and mode values for each antifungal agent. NLC‐Luliconazole (NLC‐LCZ) showed outstanding antifungal activity and consistent efficacy against all 48 tested isolates. The MIC values were highly uniform, with a MIC_50_, MIC_90_, geometric mean, and mode of 0.016 μg/mL. *LCZ* also displayed high potency, with MICs of 0.016 μg/mL for 46 isolates and 0.031 μg/mL for the remaining 2, yielding a range of 0.016–0.031 μg/mL. Like NLC‐LCZ, LCZ had a MIC_50_, MIC_90_, geometric mean, and mode of 0.016 μg/mL, demonstrating robust and uniform antifungal activity. In contrast, ITZ exhibited a much wider MIC distribution, ranging from 0.016 μg/mL to > 16 μg/mL. The MIC_90_ (8 μg/mL) indicated a notable shift toward higher resistance compared to the LCZ compounds. The geometric mean MIC was 0.659 μg/mL, while the mode was 0.063 μg/mL. TRB exhibited a broad MIC range, spanning from 0.016 μg/mL to > 4 μg/mL. The MIC_90_ was determined to be 4 μg/mL, while the geometric mean and mode MIC values were 0.293 μg/mL and 0.125 μg/mL, respectively. Overall, NLC‐LCZ and LCZ exhibited markedly greater in vitro activity against *T. indotineae* isolates than ITZ and TRB, demonstrated by their substantially lower MIC_50_, MIC_90_, and geometric mean values, along with a narrow MIC range. A Kruskal‐Wallis H test revealed a statistically significant difference among the four groups (H = 147.7, *p* < 0.0001), indicating significant variation in MIC distributions. The results showed that the NLC group was significantly different from the ITZ group (adjusted *p* < 0.0001) and the TRB group (adjusted p < 0.0001). However, no significant difference was found between the NLC and LCZ groups or between the TRB and ITZ groups (adjusted *p* > 0.9999).

**TABLE 1:** Antifungal susceptibility of 48 *Trichophyton indotineae* isolates to four antifungal drugs.

Antifungal agents	MIC (μg/mL)													MIC range	MIC_50_	MIC_90_	G mean	Mode
	0.004	0.008	0.016	0.031	0.063	0.125	0.25	0.5	1	2	4	8	16					
NLC‐ LCZ			48											0.016	0.016	0.016	0.016	0.016
Luliconazole			46	2										0.031–0.016	0.016	0.016	0.016	0.016
Itraconazole			1	1	12	3	6	2		5	11	2	5	0.016‐ > 16	0.5	8	0.659	0.063
Terbinafine			5	3	2	12	6	6	2	2	10			0.016‐ > 4	0.25	4	0.293	0.125

Abbreviations: MIC, Minimum inhibitory concentration; NLC‐LCZ, Nanostructured Lipid Carriers Luliconazole.

### 3.3 Participant Demographic Data

A total of 48 patients were included in the study, comprising 28 males (58.3%) and 20 females (41.7%). The majority of participants were aged 21–40 years (50%, *n* = 24), followed by those aged 41–55 years (37.5%, *n* = 18). Patients aged 18–20 years accounted for 12.5% of the study population (*n* = 6). Regarding occupation, housewives represented the largest group (22.9%, *n* = 11), followed by employees (16.7%, *n* = 8) and freelancers (14.6%, *n* = 7).

Clinical evaluation revealed a distinct distribution of infection locations. Because some patients presented with infection at more than one anatomical site, the total number of affected sites (*n* = 72) exceeded the number of patients (*n* = 48). Therefore, percentages related to infection sites were calculated based on the total number of affected sites rather than the total number of patients. Tinea cruris was the most prevalent presentation, affecting 44.4% of affected sites (*n* = 32), followed by tinea corporis (37.5%, *n* = 27). Less frequent manifestations included tinea pedis (8.3%, *n* = 6), tinea manuum (6.9%, *n* = 5), and tinea faciei (2.8%, *n* = 2). Notably, no cases of tinea capitis or tinea unguium were identified. Multiple‐site involvement was observed in 28.9% of patients (*n* = 15). Additionally, 14 patients (29.2%) presented with concurrent involvement of the groin and buttock regions. A pattern suggestive of intrafamilial clustering was observed, as four married couples and three families presented with infections during the same period; however, definitive confirmation of person‐to‐person transmission was beyond the scope of the present study. Sex‐based distribution patterns were evident: tinea cruris was more common among males (67.8%, *n* = 19/28), whereas tinea corporis was more frequently observed among females (55%, *n* = 11/20). Overall, the majority of infections occurred in adults aged 21–55 years, with the groin and buttock regions representing the most frequently affected anatomical sites. Demographic and clinical characteristics are summarized in Table 2 and Figure 1.

**TABLE 2:** Summary of patient demographic characteristics.

Category	Subcategory	Count	Percentage
Gender	Male	28	58.3%
	Female	20	41.7%
Age Distribution	≤ 20 years	6	12.5%
	21–40 years	24	50%
	41–55 years	18	29.16%
Occupation	Homemaker	11	22.9%
	Employee	8	16.66%
	Freelancer	7	14.58%
	Retired	4	8.33%
	Military	4	8.33%
	Student	3	6.25%
	Nurse	2	4.16%
	Other/Unspecified	9	18.75%
Clinical site of infection^a^	Tinea cruris	32	44.45%
	Tinea corporis	27	37.5%
	Tinea pedis	6	8.33%
	Tinea manuum	5	6.94%
	Tinea faciei	2	2.78%
Multiple infection sites	Patients with > 1 site	15	28.90%
Gender specific infection	Males with groin infections	19/28	67.8%
	Females with body infections	11/20	55.0%

^a^ Percentages for infection sites are calculated based on the total number of affected sites (*n* = 72), which exceeds the number of patients because some individuals had infections at multiple anatomical locations.

### 3.4 Treatment Outcomes: Clinical and Mycological Efficacy

A total of 48 patients with TRB‐resistant dermatophytosis caused by *Trichophyton indotineae* were randomly assigned to four treatment groups: ITZ alone, LCZ gel, NLC‐LCZ gel, and ITZ + NLC‐LCZ gel. The primary outcome was the rate of complete recovery, defined as resolution of all clinical signs and symptoms together with negative results on direct microscopy and fungal culture. Complete recovery rates differed significantly among groups: ITZ 0%, LCZ gel 58.3%, NLC‐LCZ 75%, and ITZ + NLC‐LCZ 83.3% (*p* < 0.01). Patients who showed clinical improvement but had persistent positive mycological findings or residual pruritus were categorized as having a partial response and were therefore not considered to have achieved complete recovery. No cases of isolated mycological cure without accompanying clinical improvement were observed.

**FIGURE 2:**
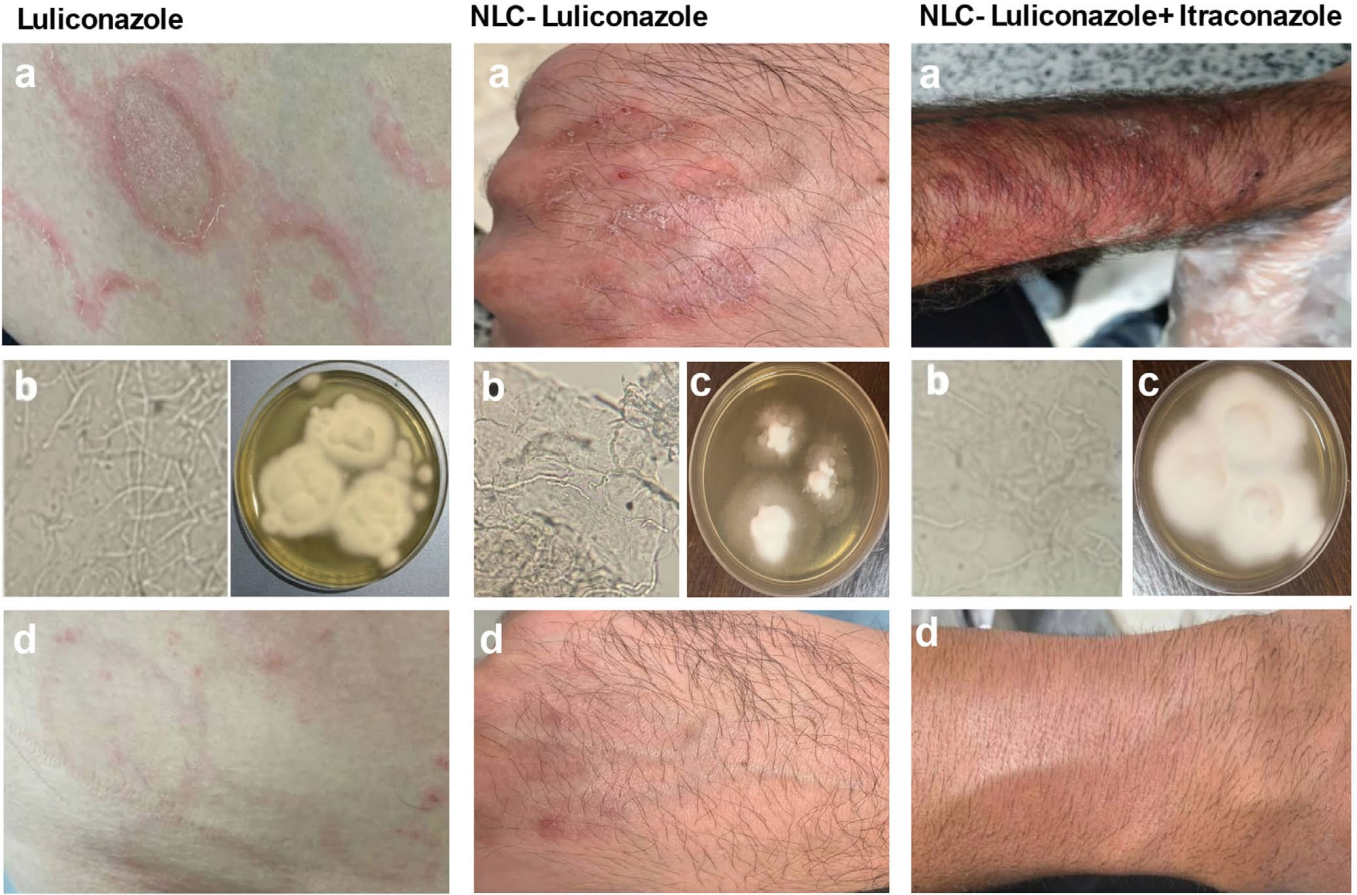
Comparative evaluation of the therapeutic efficacy of Luliconazole (LCZ), NLC‐Luliconazole (NLC‐LCZ), and the combination of NLC‐LCZ + Itraconazole (ITZ) in *Trichophyton indotineae* dermatophytosis. Representative images from the three treatment groups are shown: LCZ (left), NLC‐LCZ (middle), and NLC‐LCZ + ITZ (right). (a) Skin lesions before treatment initiation. (b) Direct microscopic examination of skin scrapings with 10% potassium hydroxide (KOH) showing septate fungal hyphae. (c) Fungal colony growth on Sabouraud Chloramphenicol Cycloheximide (SCC) agar confirming T. indotineae infection. (d) Clinical appearance of the skin after completion of treatment.

Symptom severity, including pruritus and inflammation, was highest in the ITZ group (mean scores 4.5 and 3.5, respectively) and significantly reduced in the NLC‐LCZ and ITZ + NLC‐LCZ groups (*p* < 0.01). Representative images (Figure 2) demonstrate marked lesion improvement and mycological clearance following treatment.

### 3.5 Adverse Effects

Adverse events were mild‐to‐moderate; no treatment discontinuation occurred. Incidence: LCZ gel 41.7%, NLC‐LCZ 16.7%, ITZ 16.7%, ITZ + NLC‐LCZ 0%. Most common events were mild itching and redness. Overall, 18.8% (9/48) experienced minor adverse events (Table 3).

**TABLE 3:** Clinical outcomes and adverse events across treatment groups.

Outcome/variable	ITZ (*n* = 12)	LCZ Gel (*n* = 12)	LCZ‐NLC Gel (*n* = 12)	ITZ + LCZ‐NLC gel (*n* = 12)	Effect size (95% CI)	*p*
Complete recovery (%)	0%	58.3%	75%	83.3%	RR vs. ITZ: 1.42 (1.10–1.84)	< 0.01
Mean itch score (mean ± SD)	4.5 ± 0.7	2.8 ± 0.6	1.8 ± 0.4	1.5 ± 0.5	Mean difference vs. ITZ: −3.0 (−3.6 to −2.4)	< 0.01
Mean inflammation score (mean ± SD)	3.5 ± 0.6	2.0 ± 0.5	1.1 ± 0.3	1.2 ± 0.4	Mean diff vs. ITZ: −2.3 (−2.9 to −1.7)	< 0.01
DLQI (final score), (mean ± SD)	10 ± 2	5 ± 1.5	2 ± 1	3 ± 1	Mean change vs. ITZ: −7 (−8.5 to −5.5)	< 0.001
Mild itching (AE), *n* (%)	2 (16.7%)	3 (25%)	2 (16.7%)	0 (0%)	Total: 7/48 (14.6%)	—
Mild redness/irritation (AE), *n* (%)	0 (0%)	2 (16.7%)	0 (0%)	0 (0%)	Total: 2/48 (4.2%)	—
Total patients with any AE, *n* (%)	2 (16.7%)	5 (41.7%)	2 (16.7%)	0 (0%)	Overall, AE rate: 9/48 (18.8%)	—

Abbreviations: ITZ, Itraconazole; LCZ, Luliconazole; NLC‐LCZ, Nanostructured Lipid Carriers Luliconazole.

### 3.6 Quality of Life Assessment

**FIGURE 3:**
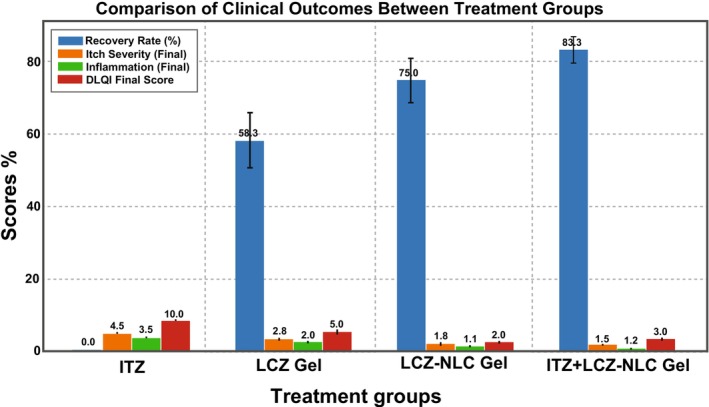
Comparison of clinical outcomes among different treatment groups: Oral ITZ, LCZ gel, NLC‐LCZ gel, and combination therapy with ITZ + NLC‐LCZ gel. Bars represent complete recovery rate (%), final itch severity score, final inflammation score, and Dermatology Life Quality Index (DLQI). Data are presented as mean ± standard deviation.

Dermatology Life Quality Index (DLQI) scores improved significantly in all groups except ITZ monotherapy. Final DLQI scores were: NLC‐LCZ 2, ITZ + NLC‐LCZ 3, LCZ gel 5, ITZ 10 (*p* < 0.001), indicating enhanced patient functioning and reduced disease burden (Table 3, Figure 3).

## 4 Discussion

The rise of severe and refractory dermatophytosis to treatment with conventional antifungal drugs in recent years has become a global concern. Initially, *T. indotineae* was confined to certain geographic areas, primarily within the Indian subcontinent. However, recent reports suggest that infections caused by *T. indotineae* are now spreading to Europe, other Asian countries, and additional continents [21]. The emergence of resistance among dermatophytes highlights the urgent need for the development of new and alternative treatment options.

In recent years, nanotechnology has garnered considerable attention, with numerous studies emphasizing its applications in microbiology and the management of antimicrobial drug resistance. In a previous study, we formulated and characterized NLC‐LCZ and evaluated the in vitro antifungal efficacy against a panel of resistant fungal strains [22] which suggested that the NLC‐LCZ formulation has the potential to be an effective topical treatment for superficial fungal infections, especially in cases with resistant strains.

The present study aimed to evaluate the efficacy of formulated NLC‐LCZ in clinical settings. All 48 patients enrolled in the study had previously received several weeks of oral TRB treatment but showed no clinical improvement. In this study, the ITZ + NLC‐LCZ group, which received oral ITZ in combination with NLC‐LCZ, demonstrated a statistically significant higher rate of recovery (83.3%). This was followed by the NLC‐LCZ group with a recovery rate of 75%, the LCZ gel group with a recovery rate of 58.3%, and the ITZ group, which exhibited a recovery rate of 0%. Although the MIC_90_ of itraconazole for the studied isolates was relatively high, the lack of clinical response in the ITZ monotherapy group may not be explained solely by antifungal resistance. In the present study, itraconazole was administered at a dose of 100 mg twice daily (200 mg/day). Itraconazole is known to exhibit considerable pharmacokinetic variability, and therapeutic drug monitoring was not performed in the present study. Therefore, subtherapeutic drug levels, host‐related factors, or variations in treatment adherence may also have contributed to the absence of clinical response. Several studies have highlighted the beneficial and synergistic effects of combining oral antifungal medications with topical treatments for managing fungal infections [23, 24, 25, 26]. This combined approach enhances therapeutic efficacy, potentially leading to faster symptom relief and improved treatment outcomes. The dual mechanism of action targets the infection both systemically and locally, which can be particularly advantageous in cases of resistant or severe infections. This strategy not only improves patient compliance but also reduces the likelihood of recurrence, making it a valuable option in the comprehensive management of fungal infections.

The NLC‐LCZ group, with a success rate of 75%, exhibited improved efficacy compared to the LCZ gel group, which had a recovery rate of 58.3%. This suggests that the nanostructured lipid carriers may enhance the therapeutic potential of LCZ in treating resistant dermatophytosis. Clinical trials and research have demonstrated that LCZ is highly effective in treating dermatophyte infections of the skin and nails, thereby providing a significantly better therapeutic option for these common fungal infections. Our previous research [22, 27], along with other studies [28] suggests that NLC systems may improve the drug‐delivery characteristics of topical antifungal agents, including enhanced solubility and skin retention. However, it should be noted that the present clinical trial did not directly evaluate pharmacokinetic parameters such as skin penetration or drug retention. Therefore, these mechanistic explanations should be interpreted cautiously and require confirmation in future pharmacokinetic and skin‐penetration studies. The analysis of symptoms at the end of 2 and 4 weeks revealed significant reductions in both itching severity and redness/inflammation in the ITZ + NLC‐LCZ group and the NLC‐LCZ group compared to the ITZ group. These findings suggest that the inclusion of NLC‐LCZ, either in combination with ITZ or alone, enhances the therapeutic efficacy in managing symptoms. The consistent improvement over the study period underscores the potential of NLC‐LCZ as a valuable component in the treatment regimen for fungal infections. The adverse effects survey revealed that the LCZ gel group experienced the highest incidence of adverse effects, specifically itching, with a total of five reported cases. In parallel with clinical improvement, Dermatology Life Quality Index (DLQI) scores showed a significant reduction in the NLC‐LCZ and ITZ + NLC‐LCZ groups, indicating a meaningful improvement in patients' quality of life compared to ITZ monotherapy. However, all adverse effects were minor and did not require discontinuation of treatment. The findings of this clinical trial indicated that the NLC‐LCZ formulation presents a promising topical treatment for resistant dermatophyte infections. This formulation may help overcome some limitations associated with LCZ, such as limited aqueous solubility and potentially suboptimal skin retention and penetration. Additionally, the NLC‐LCZ formulation was found to be safe, with minimal side effects, and was generally well tolerated by patients. However, the combination of oral ITZ with topical NLC‐LCZ demonstrated the most significant therapeutic effect. This suggests that a combination approach may enhance treatment efficacy, shorten the duration of therapy, and improve overall patient outcomes.

### 4.1 Limitations

This study has several limitations. The relatively small sample size and single‐center design may limit the generalizability of the findings. In addition, the follow‐up period was relatively short (4 weeks), which did not allow for the evaluation of long‐term outcomes, including relapse of dermatophytosis or delayed adverse effects. Considering the high relapse rates reported in infections caused by *T. indotineae*, future studies with longer follow‐up periods are necessary to more accurately assess recurrence rates and long‐term treatment outcomes. Furthermore, pharmacokinetic parameters such as skin penetration and drug retention of the NLC‐LCZ formulation were not directly evaluated in this clinical trial; therefore, the proposed mechanisms related to enhanced drug delivery should be interpreted cautiously and confirmed in future pharmacokinetic and skin‐penetration studies. Finally, a placebo‐controlled design was not considered ethically appropriate, as withholding antifungal treatment from patients with active fungal infections could lead to disease progression. Consequently, all participants received active antifungal therapy, and complete double‐blinding was not feasible due to differences in the routes of administration (oral versus topical).

## 5 Conclusion

This study demonstrates that NLC‐LCZ represents an effective and well‐tolerated topical option for the management of TRB‐refractory *T. indotineae* infections. NLC‐LCZ, either alone or in combination with oral itraconazole, achieved significantly higher complete recovery rates, greater symptom reduction, and improved quality‐of‐life outcomes compared to conventional formulations. The combination therapy of ITZ + NLC‐LCZ showed the highest clinical efficacy, indicating that a dual systemic–topical approach may be the most beneficial strategy for overcoming antifungal resistance. Given their enhanced skin penetration, improved drug retention, and favourable safety profile, NLC‐LCZ has the potential to address current limitations in the treatment of resistant dermatophytosis and warrant further large‐scale studies to confirm these findings.

## Author Contributions

**Hossein Mohammad Taghi Fam:** writing – review and editing, methodology. **Seyyed Mobin Rahimnia:** writing – review and editing, methodology, investigation. **Mahdi Abastabar:** writing – review and editing, conceptualization, methodology, validation, project administration, resources, funding acquisition. **Seyed Ali Jeddi:** methodology, writing – review and editing, writing – original draft, data curation. **Jacques F. Meis:** writing – review and editing, investigation. **Ghasem Rahmatpour Rokni:** methodology, writing – review and editing, investigation. **Robab Ebrahimi Barogh:** methodology, writing – review and editing, writing – original draft, data curation. **Armaghan Kazeminejad:** writing – review and editing, methodology. **Eisa Nazar:** writing – review and editing, investigation, software, formal analysis. **Pedram Ebrahimnejad:** writing – review and editing, investigation. **Zahra Yahyazadeh:** writing – review and editing, methodology. **Nasim Gholizadeh:** writing – review and editing, methodology, investigation. **Abdullah M. S. Al‐Hatmi:** writing – review and editing, investigation. **Mohsen Nosratabadi:** writing – review and editing, writing – original draft. **Ahmed Al‐Harrasi:** writing – review and editing, investigation. **Mohammad Taghi Hedayati:** writing – review and editing, investigation. **Iman Haghani:** writing – review and editing, methodology, investigation. **Abbas Raeisabadi:** writing – review and editing, methodology. **Javad Javidnia:** writing – review and editing, investigation, methodology, software, visualization.

## Funding

This study was supported by Mazandaran University of Medical Sciences, Sari, Iran, Grant/Award Number: 20072.

## Ethics Statement

The current study was approved by the Ethics Committee of the Mazandaran University of Medical Sciences (IR.MAZUMS.REC.1403.048) on 11 March 2024. Additionally, the trial was registered with the Clinical Trials Ethics Committee (Registration Number: IRCT20240711062393N1).

## Consent

Written informed consent was obtained from all participants involved in the study.

## Conflicts of Interest

The authors declare no conflicts of interest.

## Supporting information


**Table S1:** Semi‐quantitative clinical scoring system used for efficacy assessment.

## Data Availability

The data that supports the findings of this study are available in the Supporting Information of this article.
