# Supplementary material for: Efficacy Evaluation of Luliconazole‐Loaded Nanostructured Lipid Carriers in Treatment‐Resistant Dermatophytosis: A Randomized Clinical Trial
Source: Mycoses. 2026 Jun 8;69(6):e70196. doi: 10.1111/myc.70196 (PMC13245194; doi:10.1111/myc.70196)
Supplement: Supplementary file 1 — Table S1: Semi‐quantitative clinical scoring system used for efficacy assessment. [file MYC-69-e70196-s001.docx]

**Supplementary Table S1.** Semi-quantitative clinical scoring system used for efficacy assessment

| **Clinical parameter** | **Score 0** | **Score 1** | **Score 2** | **Score 3** | **Score 4** | **Score 5** |
| --- | --- | --- | --- | --- | --- | --- |
| **Lesion size** | No visible lesion | Minimal involvement | Mild involvement | Moderate involvement | Extensive involvement | Very extensive involvement |
| **Pruritus (itching)** | None | Very mild | Mild | Moderate | Severe | Very severe |
| **Inflammation (erythema/edema)** | None | Very mild erythema | Mild erythema | Moderate erythema | Severe erythema | Very severe erythema with marked inflammation |
| **Scaling** | None | Very mild | Mild | Moderate | Severe | Very severe |
| **Patient satisfaction** | Completely satisfied | Satisfied | Slightly satisfied | Neutral | Dissatisfied | Very dissatisfied |
